# Short- and medium-term cost effects of non-indicated thyroid diagnostics: empirical evidence from German claims data

**DOI:** 10.1007/s10198-021-01382-1

**Published:** 2021-11-22

**Authors:** Lucas Hafner, Valeria Biermann, Susann Hueber, Ewan Donnachie, Thomas Kühlein, Harald Tauchmann, Johanna Tomandl

**Affiliations:** 1 Universität Erlangen-Nürnberg, Nuremberg, Germany; 2 Universitätsklinikum Erlangen, Erlangen, Germany; 3 Kassenärztliche Vereinigung Bayerns, Munich, Germany; 4 Professur für Gesundheitsökonomie, Fachbereich Wirtschafts- und Sozialwissenschaften, Universität Erlangen-Nürnberg, Findelgasse 7/9, 90402 Nuremberg, Germany; 5 CINCH—Health Economics Research Center, Essen, Germany; 6 RWI—Leibniz Institut für Wirtschaftsforschung, Essen, Germany

**Keywords:** Matching, Health cost, Non-indicated ultrasound, Thyroid anomalies, I11, I18

## Abstract

This paper contributes to the discussion of whether non-indicated ultrasound examinations of the thyroid gland contribute to overtreatment and excess health care expenditures. Using two sources of claims data from Germany, we analyzed data from patients who underwent a TSH blood test which is the initial diagnostic measure to check for possible presence of thyroid dysfunction. In a matching analysis, we compared health costs of two groups of patients. One consisted of patients who underwent an early thyroid ultrasound that according to medical guidelines—at this point—was probably not indicated. The other group consisted of patients, who underwent no ultrasound examination at all or later in the course of the disease, making probable a correct indication. Both groups were made comparable by the means of a matching procedure. Average thyroid-specific health costs were substantially higher for the first group in the quarter in which the ultrasound examination took place. Some deviation in these specific costs persisted over a substantial period of time, with drug expenditures exhibiting the biggest difference. If, however, total health costs were considered, difference in costs was only found in the initial quarter. We conclude that non-indicated ultrasound examination of the thyroid gland may have some moderate effects on thyroid-specific costs. Yet the data do not suggest that long-lasting overtreatment and excess health expenditures are initiated by non-indicated ultrasound in Germany.

## Introduction

In recent years, the increasing incidence of thyroid cancer has repeatedly been discussed in the literature. Several studies from different countries document this increase [1–5]. According to a study by Vacarella et al. [6], the age-standardized incidence of thyroid cancer of women in the United States increased from 9.1 cases per 100,000 inhabitants in 1988–1992 to 19.2 cases per 100,000 inhabitants in 2003–2007. A similar change can also be observed in European countries, where for instance the incidence of thyroid cancer of women in France increased from 6.9 to 16 cases per 100,000 inhabitants over the same period. The most significant change in the incidence of thyroid cancer was probably observed in South Korea. There has been an increase from 12.2 to 59.9 cases per 100,000 inhabitants. However, in terms of absolute numbers thyroid cancer mortality has hardly changed or even decreased [1–3, 7]. This puzzle can partly be explained by the majority of cases detected being so-called papillary carcinomas, which mostly have a favorable prognosis. Patients with papillary cancer have a 10-year survival rate of 80–90% [8, 9].

The phenomenon of an increasing incidence combined with almost stable mortality is widely accepted as a sign of overdiagnosis, which can be defined as “the detection of indolent pathology where treatment cannot provide benefit”[10],^1^ while overtreatment means “that a treatment provides no benefit for the diagnosed condition” [11]. A high number of thyroid nodules suspicious of cancer turn out to be benign postoperatively and therefore their removal dispensable in retrospect. In Germany having the highest rates of thyroidectomies worldwide the ratio of malign to benign nodules as diagnosed histologically after their removal is 1:15, doing harm also to those without cancer [12]. Both, overdiagnosis and overtreatment which includes but goes beyond the treatment of overdiagnosed conditions, are summarized under the catchword medical overuse [13].

One of the consequences of non-indicated diagnostics is the risk of so-called cascade effects, defined as processes that, once initiated, proceed step-by-step until their seemingly almost inevitable outcome [14]. Ultrasound examination of the thyroid gland may act as a starting point of such a cascade initiating a process of further testing and controls finally ending in unnecessary thyroidectomies and radioiodine therapy. The increased sensitivity of diagnostic tests (e.g. ultrasound with 13 instead of 7.5 megahertz) contributes significantly to overdiagnosis, because there is a high risk of identifying benign nodules and non-fatal carcinomas, which are then further diagnosed and treated [15].

Identifying such cascades, in particular the factors by which they are triggered, is an important topic to be researched. Preventing such cascades from starting and accelerating can contribute to a better allocation of limited resources in a health system and improve patients’ wellbeing [16]. Since the number of persons diagnosed with thyroid nodules and thyroid cancer continues to rise and the duration of treatment is usually substantial due to the majority of thyroid carcinomas being not metastatic [17], better knowledge of the associated costs is required. Overdiagnosis and overtreatment are potential problems from an ethical as well as from an economic perspective. Medical services that do not provide any or only little benefit to patients or the harms of which exceed their potential benefits are wasting money that is urgently needed in other places [18].

As of now, the monetary costs of thyroid nodules and cancer care caused by medical overuse in Germany are not comprehensively evaluated. The contribution of this paper is to provide approximate answers to the questions whether questionable early thyroid ultrasound leads to cascade effects in the care of affected patients and what effects these avoidable cascades have on expenditures in the health care system. The aim of this paper is, therefore, to get more insights into the costs of medical overuse.

## Methods

### Data, sample selection and matching

#### Data

The analysis is based on two sources of quarterly administrative billing data from Germany, for the years 2012 to 2016. Two data sets have been analyzed: one data set was provided by the Bavarian Association of Statutory Health Insurance Physicians (Kassenärztliche Vereinigung Bayerns, KVB), which represents all physicians and psychotherapists licensed for outpatient care, under the roof of the social health insurance, in the state of Bavaria. All ambulatory care physicians send their reimbursement claims quarterly to their corresponding Association of Statutory Health Insurance Physicians. The data set contains information about patient characteristics (age, sex, ICD diagnosis, billing codes for medical and diagnostic treatment) and physicians’ characteristics, e.g. whether it is a group practice or single handed. The data can also be linked to information of the region as for example density of population according to the Federal Office for Building and Regional Planning or deprivation [19]. These data hence do not comprise information on inpatient treatment, results of medical tests or procedures and they are confined to patients from a single federal state. Yet, since the data comprises (almost) all publicly insured outpatient cases in Bavaria, the second biggest federal state of Germany, the number of observations is still very large. The second data set was provided by the Corporation for Efficiency and Quality in Health Insurance (Gesellschaft für Wirtschaftlichkeit und Qualität bei Krankenkassen, GWQ). The GWQ is owned by—and provides data services to—25 of in particular company-based^2^ sickness funds. However, only ten health insurance companies agreed to participate in the project. Accordingly, only the data from these funds could be used. Like the KVB data, the GWQ data include information about both patients and physicians (see above). The GWQ data are not limited to outpatient care but also include information on inpatient stays, prescribed drugs, and sick pay. Moreover, since company-based sickness funds operate nationwide, the data cover Germany as a whole.

The populations from which the two data sets originate hence differ. Besides the geographic confinement of the KVB data, self-selection into company-based health insurance is an issue. Individuals insured with company-based funds on average are younger [20] and healthier [21] than the overall population. Neither of the data sets used includes privately insured individuals. Though some patients may be included in both the KVB and the GWQ data, the number of observations for which this applies is likely to be small. The intention to use two different sources of observations is hence not to match information on the individual patient level^3^ but to compare results across different data sources that vary in several dimensions as described above and to exploit the relative advantages of the two data sources, which in terms of size and self-selection issues are with the KVB data and in terms of comprehensiveness of the cost information are with the GWQ data.

### Sample description

Both data sources comprise patients who were 18 years or older and received a TSH test (EBM code 32101) for the first time in 2012. Patients who received any thyroid-specific tests or diagnoses in 2010 or 2011 have been excluded.^4^ Patients had to be insured throughout 2010 to 2012, while gaps of a maximum of 30 days were allowed. Patients aged 110 years and older and those who lacked unique information regarding gender and date of birth were excluded. This also applies to individuals for whom implausible diagnoses were reported.^5^

The sample taken was then split into two groups: a so-called ‘observation group’ and a ‘control group’. We used this terminology to emphasize the quasi experimental design of the analysis that we aimed to achieve through matching (described below). The analysis was based on observational data and did not involve any randomized treatment or intervention that was under the control of the experimenter. Patients in the observation group had an initial TSH test in 2012^6^ and a thyroid ultrasound (EBM code 33012) within four subsequent weeks (0–28 days). The control group consisted of patients who received a TSH test in 2012 but received either no ultrasound at all or 28 days after the TSH test at the earliest. The definition of the two groups is based on the recommendations of the guideline of the German College of General Practitioners and Family Physicians (DEGAM) [22]. The guideline recommends in case of a first abnormal TSH test and an inconspicuous anamnesis, a second TSH test to be performed. In case of a second abnormal TSH test, further laboratory tests (ft4, ft3, antibodies) are recommended. According to international guidelines an ultrasound of the thyroid gland would only make sense in case of a palpably enlarged goiter, palpable thyroid nodules or lymph nodules or in case of hyperthyroidism in the absence of serological markers for thyroiditis or Graves’ disease, because then it might be caused by an autonomous adenoma [23, 24]. As all these findings are rare, it can be assumed that the vast majority of early ultrasound examinations have to be regarded as non-indicated, constituting an unjustified screening. Patients in the observation group with an initial diagnosis of hypo- or hyperthyroidism in the uptake quarter were excluded, as in these cases the early use of an ultrasound might have been reasonable.

The considered outcomes are all cost measures that are calculated from the billing information available in the two data sources used. Since outpatient services are reimbursed on a quarterly basis, all cost measures refer to costs per quarter. In other words, the unit of time considered in the analysis is the quarter of a year.

### Matching

We applied propensity score matching to establish comparable baseline conditions for the observation and control groups to be able to measure the cost effects of the (potentially redundant) ultrasound examination. The propensity score, which equals the likelihood of receiving an ultrasound within 28 days of the initial TSH test, was calculated via logistic regression.^7^ Our matching variables were based on information gathered prior to the initial TSH. It included socioeconomic information such as the patient’s age, gender and place of residence. Additionally, we included medical variables like the reason for physician consultation—in particular whether a TSH test was accompanied by a specific diagnosis i.e. whether a relevant complaint existed or whether the TSH was determined only routinely,^8^ and the number of applicable risk groups according to the grouper suggested by InBA (Institut des Bewertungsausschusses) as an indicator for multi-morbidity [25]. We opted for one-to-one nearest neighbor matching. That is, each patient from the observation group was assigned one patient from the control group, whose probability of receiving the treatment was most similar. The observed outcome (costs) of this individual, hence, served as the estimated counterfactual outcome of the treated patient i.e. as an estimate of the health cost that would have been observed if the patient had not undergone a non-indicated ultrasound examination.

Regarding the data from the KVB, 665,126 patients entered the matching process. After matching, 68,862 patients were part of the observation and control group, respectively. Regarding the GWQ data 132,613 patients entered the matching process. After propensity score matching, 11,306 patients were included in the observation and the control group, respectively.

Table 1 shows the mean values of the covariates in the year 2012 for the observation group as well as the control group before the propensity score matching (columns one and two) and after the propensity score matching (columns three and four). We evaluated matching quality by means of the standardized bias in percent [26]^9^ Columns five and six of Table 1 show the standardized differences between the observation and control group before and after propensity score matching. The matching successfully established similarity in terms of means of our observable variables across our observation and control groups. It reduced the standardized bias of all covariates substantially. The largest standardized bias was only at 0.85 for the KVB data, while 3.58 was the largest standardized bias for the GWQ data. All post-matching standardized biases were thus far below the rule of thumb threshold of five percent [27].

**Table 1:** Pre-treatment means of observation and control group and standardized bias

	Unmatched		Matched		Standardized bias %		
	Observation	Control	Observation	Control	Unmatched		Matched
GWQ data							
Age	45.75	48.01	46.51	45.97	14.08		3.58
Female	0.60	0.48	0.59	0.59	23.04		0.25
No indication	0.62	0.61	0.62	0.63	2.30		1.11
InBA grouper	6.50	7.27	6.33	6.30	14.97		0.64
N	11,453	121,160	11,306	11,306			
KVB data							
Age	47.60	49.20	47.60	47.70	9.20		0.61
Female	0.62	0.54	0.62	0.62	16.05		0.00
No indication	0.59	0.58	0.59	0.59	1.62		0.00
# InBA = 0	0.11	0.08	0.11	0.11	7.91		0.65
# InBA = 1	0.06	0.05	0.06	0.06	4.39		0.85
# InBA = 2	0.07	0.07	0.07	0.07	2.38		0.39
# InBA > = 3	0.76	0.80	0.76	0.77	9.48		0.24
N	68,862	596,264	68,862	68,862			

### Presentation of results and statistical inference

To allow straightforwardly compare how the mean health costs in both groups evolved over time, we present the results in the form of bar plots. The figures below all have the same structure. The vertical axis represents the health costs while the horizontal axis represents the respective quarter since the initial TSH test, ranging from quarter 0 (the quarter where the initial TSH test was applied) to quarter 19. The bars in light grey depict the average cost of the control group, while the bars in dark grey represent the average cost of the observation group. Each bar is accompanied by a 95% normal distribution^10^-based confidence interval. To decide by eyeballing whether observation and control group exhibit cost differentials that can most likely not be attributed to sampling error, we examine whether the confidence intervals do not overlap. This graphically easily depictable approach does not one-to-one correspond to a *t* test on equal group mean costs but is more conservative, since the confidence bands may overlap despite the *t* test rejecting the null, but not the other way round [28]. The results of the corresponding formal *t* tests are documented—together with the precise values of the group-specific costs—in the Appendix, see Tables 6–17.

### Robustness check

The main specification of the empirical analysis rests on the assumption that an ultrasound within four weeks after a TSH test is in the vast majority of cases unnecessary or at least is not medically indicated. However, the data does not include information regarding the results of the TSH test. This is why we cannot rule out that TSH tests of patients in the observation group yielded more frequently suspicious results, making the physician consider an ultrasound to be reasonable although it was not yet indicated by the criterion defined above. To be able to rule out that the ultrasound was performed on the basis of an abnormal TSH test result, in a robustness check we changed the definition of the observation and control group as follows: the observation group includes only cases where both a TSH test and thyroid ultrasound were performed on the same day. The control group, on the other hand, also requires a TSH test to be performed, but a thyroid ultrasound was not performed on the same day, but one day after the TSH test at the earliest. Since in the analysis the TSH test and the ultrasound were performed simultaneously in the observation group, the result of the blood test cannot have triggered the ultrasound examination. This alternative empirical analysis is confined to the KVB data and uses, just like the main specification, a matched sample. The sample that enters the matching procedure is the same as for the main specification. Yet the alternative definition of the observation group reduces its size to 36,120 observations. In consequence, the robustness check is based on these observation and the same number of matching partners from the control group. Information for the alternative matching that parallels what is reported for the main specification in Table 1 is reported in the Appendix (Table 18).

## Results

### Outpatient costs

We started with comparing outpatient costs for the observation and control group based on data from the KVB and GWQ, respectively. In doing this, we distinguish between thyroid-specific costs (EBM codes that refer to thyroid-specific outpatient services as listed in Table 2 in the appendix) and total costs. The term total costs, hence, does not refer to aggregating costs over different sectors (outpatient, inpatient etc.) but means that cost are considered irrespective of whether or not they are thyroid-specific. If costs are aggregated over different sectors, we refer to this as overall costs (see subsection overall costs).^11^ By design both patients in the observation and the control group had to have received a TSH test in quarter 0 and thus visited a physician. Consequently, the costs in this quarter were naturally higher compared to other quarters, in which neither members of the observation group nor members of the control group had even necessarily visited a physician. The remarkable difference in cost of the initial quarter compared to the following quarters is hence partly an artifact of the design of the analysis.

### Thyroid-specific outpatient costs

**Fig. 1:**
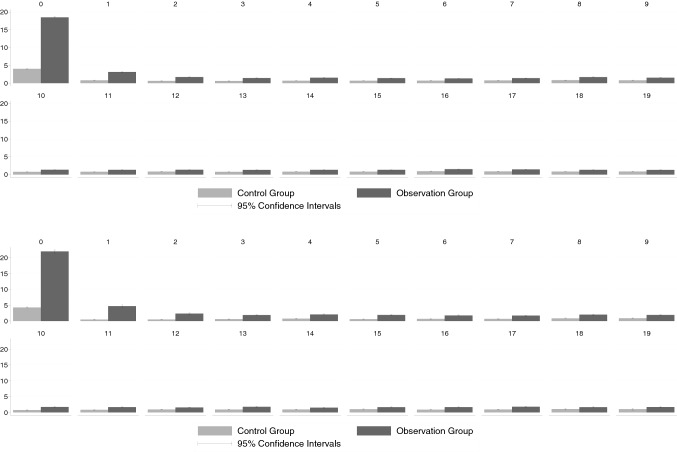
Thyroid-specific outpatient costs. Upper panel **a** KVB data, lower panel, **b** GWQ data; see Appendix Tables 6 and 7 for precise numerical values

Figure 1 displays the development of thyroid-specific outpatient costs. As already noted above, we see significantly higher thyroid-specific outpatient costs for the observation group in the quarter in which the TSH test was performed. The difference between the observation group and the control group is slightly higher than the pure cost of the ultrasound itself.^12^ This suggests that, on average, further thyroid-specific examinations were carried out in the observation group. In the following quarters, a significant cost difference between the observation group and the control group persists. Yet, given that thyroid-specific costs are on average less than 5 € in the subsequent quarters, the deviation in costs between groups is of little relevance. With respect to thyroid-specific outpatient costs the pattern is almost the same for the KVB and GWQ data. Yet, the much larger size of the KVB sample results in much narrower confidence intervals compared to the GWQ data.

### Total outpatient costs

**Fig. 2:**
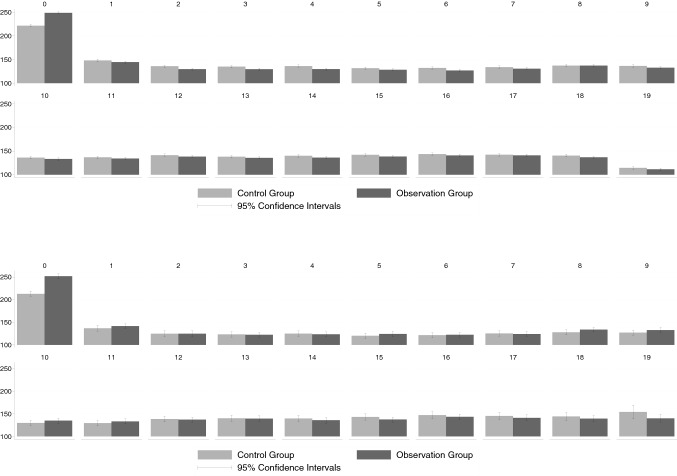
Total outpatient costs. Upper panel **a** KVB data, lower panel **b** GWQ data; see Appendix Tables 8 and 9 for precise numerical values

When considering the total outpatient costs (displayed in Fig. 2), we see—as for the thyroid-specific costs—that the costs in quarter 0 were significantly higher in the observation group compared to the control group. Yet, this cost difference exceeded the thyroid-specific cost differences. This finding suggests that patients who got the possibly non-indicated ultrasound also received more services that were not directly thyroid-related. However, this difference only occurs in quarter 0 and almost completely disappears in the following quarters. This pattern, once again, is the same for the analysis based on the KVB and the GWQ data.

### Inpatient costs

To address possible effects of the possibly non-indicated ultrasound on the costs of inpatient care we had to focus on the GWQ data since the KVB data lack information on inpatient treatment and the associated costs. Based on the analysis of the data from GWQ, the pattern of how inpatient treatment costs differentially evolve for the two groups is similar to that for outpatient treatment costs.

**Fig. 3:**
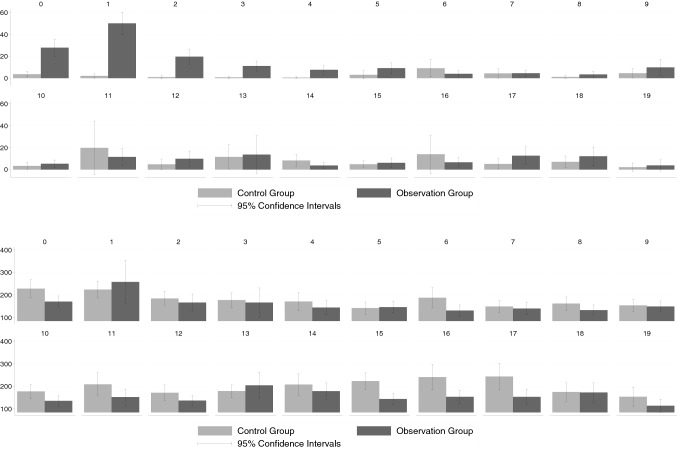
Inpatient costs (GWQ data). Upper panel **a** thyroid-specific costs, lower panel, **b** total costs; see Appendix Tables 10 and 11 for precise numerical values

Figure 3(a) shows the development of thyroid-specific inpatient costs.^13^ The average thyroid-specific costs in quarter 0 were clearly higher in the observation group, i.e. the confidence intervals do not overlap. The difference increased further in the quarter after the TSH test and the ultrasound, caused by an increase in the average cost of the observation group. In the second quarter after the initial TSH determination the costs fell back below the level of quarter 0, although the difference between the two groups was still significant. Even three and four quarters after the initial TSH test, the difference between the observation group and the control group remained significant, even though the difference continued to decrease. After five quarters of the initial TSH determination, we could no longer detect any significant difference in inpatient costs.

### Total inpatient costs

An overview of the development of total inpatient costs is shown in Fig. 3(b). Similar to the outpatient results, inpatient thyroid-specific costs account for only a very small part of total inpatient costs. In contrast to outpatient total costs, however, there is not even a significant difference between our two groups in inpatient total costs in quarter 0. In terms of the point estimates, inpatient costs seem to be even higher for the control group in later quarters. Given the rather noisy estimates and in consequence rather wide confidence bands, this may be attributed to sampling error.

### Pharmaceutical costs

**Fig. 4:**
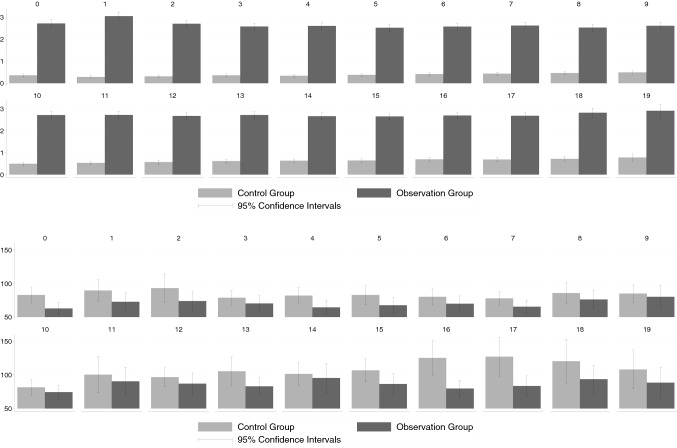
Pharmaceutical costs (GWQ data). Upper panel, **a** thyroid-specific costs, lower panel, **b** total costs; see Appendix Tables 12 and 13 for precise numerical values

Unlike the cost measures considered so far, we find an almost constant difference over time between the observation group and the control group with regard to thyroid-specific pharmaceutical costs.^14^ The average thyroid-specific pharmaceutical costs of the observation group were significantly higher than those of the control group. Concerning the total costs for pharmaceuticals, no significant differences could be identified as a result of the presumably unnecessary ultrasound. This corroborates our earlier finding that even if some effects on thyroid-related costs exist, these specific costs are too small compared to the total health costs to significantly matter (see Fig. 4).

### Overall costs

Figure 7 depicts group-specific mean overall healthcare costs that is the sum of outpatient, inpatient, and pharmaceutical costs. Since the latter two cost categories are not part of the KVB data, the comparison of overall cost is only possible for the GWQ data. Considering only thyroid-specific cost, the overall cost differentials roughly mirror the pattern found for the inpatient cost (Fig. 3a). While clearly higher costs are observed for the observation group in the two quarters that directly follow the TSH test, the cost differential shrinks in subsequent quarters an vanishes in terms of statistical significance after six quarters. If the analysis is not confined to thyroid-specific costs (Fig. 7b) the analysis provides little evidence for systematically higher overall health costs in the observation group. This also mirrors the earlier results regrading inpatient costs (Fig. 3b) including the finding, that average costs are even lower in the observation group some years after the TSH tests.

### Robustness check

**Fig. 5:**
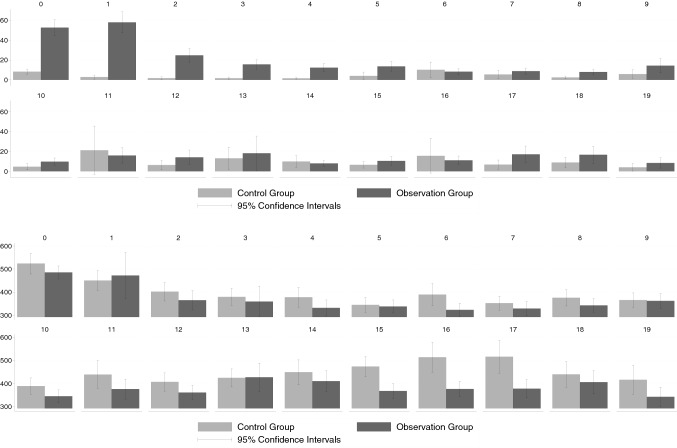
Overall costs (GWQ data). Upper panel, **a** thyroid-specific costs, lower panel, **b** total costs; see Appendix Tables 14 and 15 for precise numerical values

**Fig. 6:**
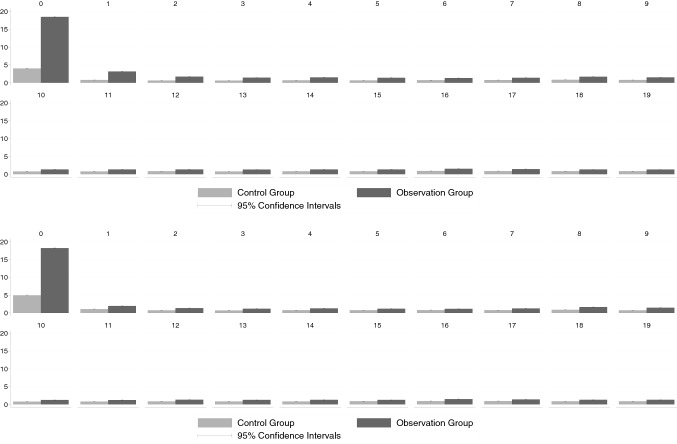
Thyroid-specific outpatient costs. Upper panel, **a** KVB data, lower panel, **b** KVB alternative matching; see Appendix Table 16 for precise numerical values

**Fig. 7:**
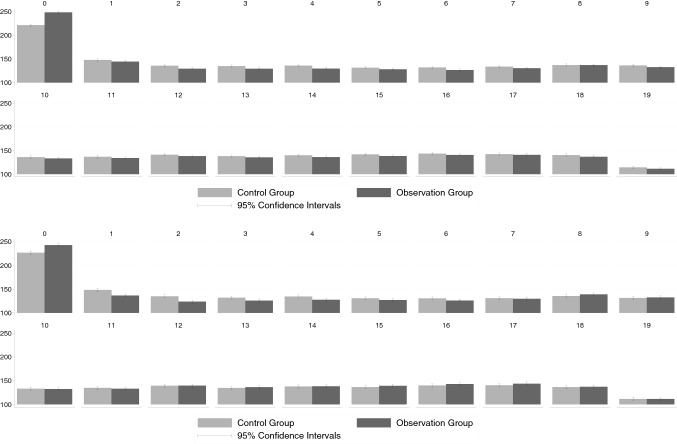
Total outpatient costs. Upper panel, **a** KVB data (same information as in Fig. 2(a)), lower panel, **b** KVB alternative matching; see Appendix Table 17 for precise numerical values

Figures 5 and 6 compare the results of the alternative design, in which the observation group consisted only of patients who received the TSH test and the thyroid ultrasound on the same day, to the previously presented results, which are based on the original treatment definition. In this comparison, we focused on the differences in thyroid-specific outpatient costs and total outpatient costs, respectively, that were found in the KVB data. The alternative definition of observation and control group hardly changed our results. Again, there was a difference in thyroid-specific costs that was still significant several quarters after the original TSH test. In terms of total costs, the picture was very similar to our main specification. In the quarters following the original examination, the ultrasound did not lead to a cascade that would be reflected in increased costs. We hence conclude that the pattern of how health costs evolved over time for the two groups was not driven by the unobserved results of the TSH blood test (see Fig. 7).

## Discussion

The results do not suggest that the presumably unnecessary ultrasound examination of the thyroid gland generally leads to a long-term increase in overall inpatient, outpatient or pharmaceutical costs. Hence the ultrasound examination does not seem to frequently act as a trigger of treatment cascades medicalizing patients and leading to a major waste of financial resources.

The picture is somewhat different if the focus is on costs specific to thyroid-related treatment. Here we see a significant short-term effect on both outpatient and inpatient costs. The latter finding may point to the ultrasound examination revealing abnormalities which were immediately examined in an inpatient setting or which led to surgeries on the thyroid gland within approximately one year. One possible explanation is that early application of ultrasound may indeed result in immediate hospital treatment that is not just earlier but additionally carried out compared to patients who received a—according to the guidelines—properly timed ultrasound examination or no ultrasound examination. This explanation is in line with the hypothesis that treatments of thyroid anomalies actually carried out, surgeries in particular, are frequently unnecessary since numerous patients would not have suffered from any disorders related to these anomalies probably until he or she dies from an unrelated disease. An alternative explanation is, however, that—even in the matched sample—an immediate ultrasound is selective in the sense that physicians do not stick to the guidelines if they—for unobserved reasons—think that further treatment might be required [29].

Moreover, costs for thyroid-specific pharmaceuticals were significantly higher in the observation group. This difference could still be observed several years after the ultrasound examination took place. Hence this result provides some support for the hypothesis that over-diagnosing thyroid anomalies may result to some extent in thyroid-related overtreatment and in consequence excess expenditures. An alternative explanation is that physicians who do not stick to medical guidelines with regard to the timing of the ultrasound examination were also less reluctant in prescribing drugs to treat possibly thyroid-related symptoms. Anyway, the share of thyroid-specific costs of total costs is relatively small. Therefore, the thyroid-specific cost effects we see in the data are of little importance to total health expenditures. Our results are against what we had expected from the literature. The steep rise of the incidence of papillary cancer of the thyroid gland, as shown in the iconic graph in Ahn’s et al. paper, was attributed to ultrasound screening of the thyroid gland as practiced routinely in South Korea [2]. Also studies from other countries suggested ultrasound screening as the reason for this rise [4, 6]. Given the high prevalence of thyroid nodules in the population and the high number of thyroidectomies in Germany in comparison to other European countries [12], we would have expected a far higher number of cascades of medical procedures with a corresponding rise in expenditures following an initial non-indicated ultrasound examination. The rise of cancer diagnoses should at least have been accompanied by a rise of diagnoses of nodular goiter.

### Limitations and strengths

Routine data mostly have the problem that the coding quality of the diagnoses has limitations [30]. A further limitation was that the data did not contain the results of diagnostic tests and the data do not provide clinical information. Therefore, it is impossible to exactly define which ultrasound examination was necessary and which not. A strength of our study can be seen in the high number of cases examined, which allows for a matching procedure that is picky in finding good matching partners. Furthermore, carrying out the analysis on basis of two data sets that complement each other with respect to the information they include can is also a strength. Therefore, it is improbable that procedures which were applied in reality were not captured in our data. The empirical analysis rets on propensity score matching which is subject to inherent limitations. While successful matching makes to considered groups comparable in terms of observables it is in principle possible that observation and control remain very different in terms of unobserved factors. Moreover, external validity is always limited for matching analyses since the effect of interest is only identified for that part of the papulation where the distribution of the propensity score overlap for the considered groups. Limited generalizability to the entire population applies in particular to matching designs like the one used in the present analysis that estimate the ATT (average treatment effect on the treated) as this design intentionally focusses on the average effect in the observation group but not the population in general [27].

## Conclusion

The data did not show the expected cascade of medical procedures after an initial unnecessary ultrasound of the thyroid gland. In consequence, the resulting costs effects are small, especially if seen as a fraction of total health care expenditures.

## Figures and Tables

**Fig. 8:**
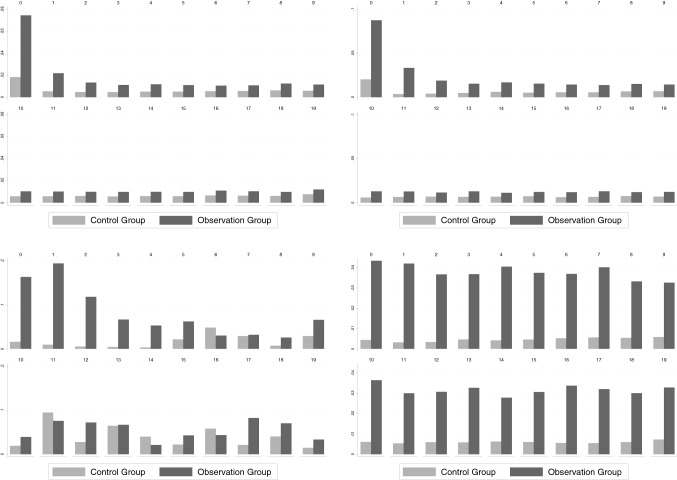
Share of thyroid-specific costs in total costs (by line). **a** Outpatient costs KVB data, **b** outpatient costs GWQ data, **c** inpatient costs GWQ data, **d** pharmaceutical costs GWQ data

## Data Availability

The manuscript presents empirical analyses that use proprietary patient-level data, which cannot be made available by the authors for data protection reasons. Researchers who want to replicate our analyses need to apply for the data at the Bavarian Association of Statutory Health Insurance Physicians (Kassenärztliche Vereinigung Bayerns, KVB) and the Corporation for Efficiency and Quality in Health Insurance (Gesellschaft für Wirtschaftlichkeit und Qualität bei Krankenkassen, GWQ).

## Appendix A

**Table 2:** Exclusion criteria

Thyroid-specific outpatient services	
2340	Thyroid puncture
17320	Quantitative and qualitative scintigraphic examination of the thyroid gland
17321	Radioiodine two-phase test
17370	Radioiodine therapy for thyroid disorders, including necessary control measurements
32101	Thyrotropin (TSH)
32320	Free thyroxine (fT4)
32321	Free triiodothyronine (fT3)
33012	Sonographic examination of the thyroid using B-mode procedure
34322	CT examination of the soft tissues of the neck
34422	MRT examination of the soft tissues of the neck, HWK 1 to HWK 7
Thyroid-specific outpatinet diagnosis	
E01	Iodine-deficient thyroid diseases and related conditions
E02	Subclinical iodine deficiency hypothyroidism
E03	Other hypothyroidism
E04	Other non-toxic struma
E05	Hyperthyroidism [thyrotoxicosis]
E06	Thyroiditis
E07	Other thyroid diseases
E89	Endocrine and metabolic disorders according to medical measures, not classified elsewhere
C73	Malignant neoplasm of the thyroid gland
D34	Benign neoplasm of the thyroid gland
D44.0	Reproduction of unsafe or unknown thyroid behavior
R94.6	Abnormal results of thyroid function tests
Thyroid-specific inpatient services	
14062	Percutaneous (needle) biopsy of the thyroid gland
14072	Thyroid percutaneous biopsy controlled by imaging techniques
15820	Biopsy of thyroid gland through incision
3030	Complex Differential Diagnostic Ultrasound with Contrast Agent
3034	Complex differential diagnostic ultrasound by Tissue Doppler Imaging [TDI] and deformation analysis of tissue [Speckle Tracking]
3035	Complex differential diagnostic ultrasound of the vascular system with quantitative evaluation
3036	Complex differential diagnostic ultrasound of soft tissue tumors with quantitative measurement
3201	Native computer tomography of the neck
3221	Computer tomography of the neck with contrast medium
3701	Scintigraphy of the thyroid gland
3753	Positron emission tomography with computer tomography (PET/CT) of the whole body
3754	Positron emission tomography with computer tomography (PET/CT) of the entire body stem and head
5060	Thyroid incision
5061	Hemithyroidectomy
5062	Other partial thyroid resection
5063	Thyroidectomy
5064	Operations on the thyroid gland by sternotomy
5069	Other thyroid and parathyroid surgeries
5303	Laryngectomy
8531	Radioiodine therapy

**Table 3:** Implausible diagnosis which lead to an exclusion from the sample

	ICD-Code (each including all subcategories)
Male	C51, C52, C53, C54, C55, C56, C57, C58, N90,
	N91, N92, N93, N94, N95, N96, N97, N98, N70,
	N71, N72, N73, N74, N75, N76, N77, O10, O11,
	O12, O13, O14, O15, O16, O40, O41, O42, O43,
	O44, O45, O46, O47, O48, O70, O71, O72, O73,
	O74, O75, O80, O81, O82, O85, O86, O87, O88,
	O89, O90, O91, O92, O94, O95, O96, O97, O98,
	O99, N99.2, N99.3, N8, O0, O2, O3, O6, C60
Female	C60, C61, C62, C63, N50, N51, N4

**Table 4:** PS estimation—logit regressions (KVB data)

	Est. Coef	S.E
Age group25–29	0*.*03	0*.*03
Age group30–34	0*.*10**	0*.*03
Age group35–39	0*.*33***	0*.*03
Age group40–44	0.33***	0*.*03
Age group45–49	0.35***	0*.*03
Age group50–54	0.32***	0*.*03
Age group55–59	0.22***	0*.*03
Age group60–64	0.18***	0*.*03
Age group65–70	0.08*	0*.*04
Age group70–74	− 0.06	0*.*04
Age group75 +	− 0.53***	0*.*04
Female	0.37***	0*.*01
OKZ09162	0.50***	0*.*08
OKZ09163	− 0.08	0.14
OKZ09171	0.34**	0.10
OKZ09172	0.81***	0*.*10
OKZ09173	0.27*	0*.*10
OKZ09174	0.48***	0*.*09
OKZ09175	0*.*86***	0*.*09
OKZ09176	0*.*42***	0*.*10
OKZ09177	0*.*38***	0*.*10
OKZ09178	0*.*70***	0*.*09
OKZ09179	0*.*30**	0*.*09
OKZ09180	0*.*23*	0*.*11
OKZ09181	0*.*20	0*.*11
OKZ09182	0*.*36**	0*.*11
OKZ09183	0*.*40***	0*.*10
OKZ09184	0*.*55***	0*.*09
OKZ09185	0*.*47***	0*.*11
OKZ09186	− 0*.*39***	0*.*12
OKZ09187	0*.*07	0*.*09
OKZ09188	0*.*41***	0*.*10
OKZ09189	0*.*73***	0*.*09
OKZ09190	0*.*53***	0*.*10
OKZ09261	0*.*64***	0*.*11
OKZ09262	0*.*54***	0*.*13
OKZ09263	1.02***	0*.*11
OKZ09271	0*.*62***	0*.*10
OKZ09272	0*.*34***	0*.*11
OKZ09273	0*.*64***	0*.*10
OKZ09274	0*.*34***	0*.*10
OKZ09275	0*.*13	0*.*09
OKZ09276	0*.*38***	0*.*11
OKZ09277	− 0*.*45***	0*.*12
OKZ09278	0*.*76***	0*.*10
OKZ09279	0*.*26*	0*.*11
OKZ09361	0*.*99***	0*.*13
OKZ09362	0*.*50***	0*.*10
OKZ09363	1.05***	0*.*12
OKZ09371	0*.*92***	0*.*10
OKZ09372	0*.*27**	0*.*10
OKZ09373	0*.*46***	0*.*10
OKZ09374	0*.*80***	0*.*10
OKZ09375	0*.*70***	0*.*09

OKZ09376	0*.*38***	0*.*09
OKZ09377	0*.*06	0*.*12
OKZ09461	− 0.11	0*.*14
OKZ09462	0*.*66***	0*.*11
OKZ09463	0*.*90***	0*.*17
OKZ09464	0*.*17	0*.*18
OKZ09471	0*.*1	0*.*11
OKZ09472	0*.*26*	0*.*11
OKZ09473	0*.*16	0*.*14
OKZ09474	0*.*53***	0*.*10
OKZ09475	0*.*47***	0*.*13
OKZ09476	0*.*33**	0*.*12
OKZ09477	0*.*62***	0*.*12
OKZ09478	0*.*48***	0*.*12
OKZ09479	0*.*02	0*.*13
OKZ09561	0*.*66***	0*.*13
OKZ09562	0*.*69***	0*.*10
OKZ09563	0*.*96***	0*.*09
OKZ09564	0*.*92***	0*.*08
OKZ09565	0*.*03	0*.*15
OKZ09571	0*.*42***	0*.*09
OKZ09572	0*.*67***	0*.*10
OKZ09573	1.10***	0*.*09
OKZ09574	0*.*89***	0*.*09
OKZ09575	0*.*55***	0*.*11
OKZ09576	0*.*33**	0*.*10
OKZ09577	0*.*03	0*.*12
OKZ09661	1.29***	0*.*10
OKZ09662	0*.*18	0*.*15
OKZ09663	0*.*72***	0*.*10
OKZ09671	1.44***	0*.*10
OKZ09672	0*.*21	0*.*12
OKZ09673	− 0.15	0*.*15
OKZ09674	− 0.32	0*.*17
OKZ09675	0*.*56***	0*.*12
OKZ09676	0*.*95***	0*.*11
OKZ09677	0*.*94***	0*.*10
OKZ09678	0*.*30**	0*.*11
OKZ09679	0*.*92***	0*.*09
OKZ09761	0*.*19*	0*.*09
OKZ09762	0*.*55***	0*.*12
OKZ09763	0*.*11	0*.*13
OKZ09764	0*.*06	0*.*15
OKZ09771	0*.*66***	0*.*10
OKZ09772	0*.*31***	0*.*09
OKZ09773	0*.*05	0*.*11
OKZ09774	0*.*39***	0*.*11
OKZ09775	− 0.08	0*.*12
OKZ09776	− 0.22	0*.*15
OKZ09777	− 0.24*	0*.*11
OKZ09778	0*.*29**	0*.*10
OKZ09779	− 0.09	0*.*11
OKZ09780	0*.*17	0*.*10
# InBA = 1	− 0.11**	0*.*03
# InBA = 2	− 0.21***	0*.*03
#InBA > = 3	− 0.35***	0*.*02
No indication	− 0.19***	0*.*01

****p* value < 0.001; ***p* value < 0.01; **p* value < 0.05

OKZ# denote regional indicators

**Table 5:** PS estimation—logit regressions (GWQ data)

	Est.Coef	S.E
Constant	0*.*076	0*.*371
Age	− 0*.*002***	0*.*001
Number CC	− 0*.*036***	0*.*002
Male	0*.*479***	0*.*021
OKZ01	0*.*086	0*.*224
OKZ02	− 0*.*237	0*.*382
OKZ03	0*.*290	0*.*44
OKZ04	0*.*360*	0*.*193
OKZ06	0*.*511**	0*.*203
OKZ07	− 0*.*161	0*.*291
OKZ08	− 0*.*080	0*.*320
OKZ09	0*.*118	0*.*285
OKZ10	0*.*076	0*.*182
OKZ12	0*.*039	0*.*168
OKZ13	0*.*183	0*.*155
OKZ14	− 0*.*015	0*.*17
OKZ15	− 0*.*107	0*.*264
OKZ16	0*.*179	0*.*219
OKZ17	− 0*.*186	0*.*276
OKZ18	0*.*118	0*.*195
OKZ19	0*.*040	0*.*193
OKZ20	0*.*994***	0*.*286
OKZ21	0*.*397**	0*.*185
OKZ22	− 0*.*034	0*.*208
OKZ23	0*.*192	0*.*272
OKZ24	0*.*273	0*.*222
OKZ25	− 0*.*129	0*.*308
OKZ26	0*.*443**	0*.*176
OKZ27	0*.*291*	0*.*156
OKZ28	0*.*357***	0*.*138
OKZ29	0*.*225	0*.*312
OKZ30	0*.*770***	0*.*171
OKZ31	0*.*745***	0*.*197
OKZ32	0*.*614***	0*.*235
OKZ33	1.02***	0*.*137
OKZ34	0*.*081	0*.*183
OKZ35	0*.*693***	0*.*192
OKZ36	0*.*514***	0*.*156
OKZ37	0*.*696***	0*.*239
OKZ38	0*.*864***	0*.*157
OKZ39	0*.*823***	0*.*234
OKZ40	1.119***	0*.*156
OKZ41	0*.*99***	0*.*167
OKZ42	0*.*976***	0*.*145
OKZ44	0*.*733***	0*.*176
OKZ45	1.121***	0*.*149
OKZ46	0*.*792***	0*.*159
OKZ47	0*.*998***	0*.*149
OKZ48	0*.*659***	0*.*183
OKZ49	0*.*541***	0*.*152
OKZ50	1.191***	0*.*154

OKZ51	1.046***	0*.*154
OKZ52	0*.*499**	0*.*21
OKZ53	1.328***	0*.*161
OKZ54	0*.*751***	0*.*27
OKZ55	0*.*806***	0*.*184
OKZ56	0*.*719***	0*.*203
OKZ57	0*.*834***	0*.*266
OKZ58	0*.*836***	0*.*181
OKZ59	1.253***	0*.*167
OKZ60	0*.*793***	0*.*174
OKZ61	0*.*82***	0*.*18
OKZ63	0*.*995***	0*.*139
OKZ64	0*.*62***	0*.*144
OKZ65	0*.*823***	0*.*156
OKZ66	1.006***	0*.*182
OKZ67	0*.*437**	0*.*177
OKZ68	0*.*237	0*.*159
OKZ69	− 0*.*002	0*.*226
OKZ70	0*.*631***	0*.*156
OKZ71	0*.*663***	0*.*142
OKZ72	0*.*19	0*.*165
OKZ73	0*.*359**	0*.*146
OKZ74	0*.*51***	0*.*138
OKZ75	0*.*295	0*.*198
OKZ76	0*.*289**	0*.*138
OKZ77	0*.*32	0*.*227
OKZ78	− 0*.*038	0*.*153
OKZ79	0*.*617***	0*.*182
OKZ80	0*.*998***	0*.*145
OKZ81	1.329***	0*.*134
OKZ82	1.021***	0*.*14
OKZ83	0*.*986***	0*.*147
OKZ84	1.045***	0*.*165
OKZ85	0*.*858***	0*.*127
OKZ86	0*.*84***	0*.*132
OKZ87	0*.*688***	0*.*206
OKZ88	0*.*534***	0*.*153
OKZ89	0*.*754***	0*.*135
OKZ90	1.553***	0*.*131
OKZ91	1.154***	0*.*128
OKZ92	1.267***	0*.*137
OKZ93	1.146***	0*.*136
OKZ94	1.044***	0*.*188
OKZ95	0*.*977***	0*.*212
OKZ96	0*.*491***	0*.*16
OKZ97	1.111***	0*.*143
OKZ98	0*.*165	0*.*321
No fatigue	− 0*.*012	0*.*033
No sleep disorder	− 0*.*025	0*.*098
No dysphagia	− 1.257***	0*.*054
No hypertension	− 0*.*017	0*.*027
No weight loss	− 0*.*502***	0*.*062
No perspiration	− 0*.*850***	0*.*324

****p* value < 0.001; ***p* value < 0.01; **p* value < 0.05

OKZ# denote regional indicators

**Table 6:** Thyroid-specific outpatient costs, KVB data (estimates correspond to Fig. 1a)

	Control group		Observation group		Test of equal group means	
Quarter	Mean (95% CI)^‡^	S.E	Mean (95% CI)^‡^	S.E	*t* statistic	*p* value
0	4.03 (3.98–4.08)	0.02	18.50 (18.35–18.64)	0.07	186.486	0.000
1	0.79 (0.74–0.83)	0.02	3.15 (3.06–3.24)	0.05	44.850	0.000
2	0.63 (0.60–0.67)	0.02	1.72 (1.66–1.78)	0.03	30.681	0.000
3	0.62 (0.59–0.65)	0.02	1.44 (1.39–1.49)	0.03	27.260	0.000
4	0.68 (0.65–0.72)	0.02	1.52 (1.47–1.57)	0.03	26.957	0.000
5	0.67 (0.64–0.70)	0.02	1.40 (1.35–1.45)	0.02	24.736	0.000
6	0.72 (0.68–0.75)	0.02	1.33 (1.25–1.37)	0.02	20.295	0.000
7	0.76 (0.72–0.79)	0.02	1.40 (1.35–1.44)	0.02	21.108	0.000
8	0.85 (0.81–0.88)	0.02	1.70 (1.65–1.75)	0.03	26.747	0.000
9	0.79 (0.75–0.82)	0.02	1.52 (1.47–1.57)	0.03	23.381	0.000
10	0.78 (0.75–0.82)	0.02	1.35 (1.31–1.40)	0.02	19.482	0.000
11	0.79 (0.75–0.82)	0.02	1.34 (1.30–1.39)	0.02	18.056	0.000
12	0.85 (0.81–0.89)	0.02	1.35 (1.31–1.40)	0.02	17.026	0.000
13	0.77 (0.74–0.81)	0.02	1.32 (1.28–1.37)	0.02	18.821	0.000
14	0.82 (0.79–0.86)	0.02	1.33 (1.29–1.38)	0.02	16.893	0.000
15	0.82 (0.79–0.86)	0.02	1.35 (1.30–1.39)	0.02	17.557	0.000
16	0.93 (0.89–0.97)	0.02	1.53 (1.48–1.58)	0.03	18.449	0.000
17	0.89 (0.85–0.93)	0.02	1.45 (1.40–1.49)	0.03	17.593	0.000
18	0.86 (0.82–0.89)	0.02	1.33 (1.29–1.38)	0.02	15.933	0.000
19	0.86 (0.82–0.90)	0.02	1.33 (1.28–1.37)	0.02	15.585	0.000

^‡^Estimated 95% confidence interval for the group mean

**Table 7:** Thyroid-specific outpatient costs, GWQ data (estimates correspond to Fig. 1b)

Quarter	Control group		Observation group		Test of equal group means	
	Mean (95% CI)^‡^	S.E	Mean (95% CI)^‡^	S.E	*t* statistic	*p* value
0	4.30 (4.18–4.42)	0.06	22.01 (21.50–22.51)	0.26	66.696	0.000
1	0.47 (0.38–0.57)	0.05	4.71 (4.36–5.05)	0.18	23.270	0.000
2	0.49 (0.41–0.56)	0.04	2.34 (2.14–2.54)	0.10	17.128	0.000
3	0.59 (0.50–0.68)	0.05	1.88 (1.70–2.05)	0.09	12.715	0.000
4	0.75 (0.62–0.88)	0.07	2.07 (1.88–2.25)	0.09	11.477	0.000
5	0.61 (0.52–0.69)	0.04	1.91 (1.73–2.09)	0.09	13.032	0.000
6	0.67 (0.56–0.79)	0.06	1.76 (1.59–1.93)	0.09	10.368	0.000
7	0.68 (0.59–0.78)	0.05	1.70 (1.54–1.86)	0.08	10.799	0.000
8	0.83 (0.72–0.94)	0.06	2.01 (1.85–2.18)	0.09	11.510	0.000
9	0.86 (0.74–0.98)	0.06	1.90 (1.71–2.08)	0.09	9.248	0.000
10	0.75 (0.65–0.85)	0.05	1.72 (1.56–1.88)	0.08	10.293	0.000
11	0.82 (0.70–0.95)	0.06	1.67 (1.52–1.83)	0.08	8.320	0.000
12	0.94 (0.81–1.06)	0.06	1.56 (1.43–1.70)	0.07	6.788	0.000
13	0.91 (0.78–1.03)	0.06	1.78 (1.60–1.95)	0.09	7.905	0.000
14	0.93 (0.81–1.05)	0.06	1.51 (1.37–1.65)	0.07	6.035	0.000
15	1.02 (0.86–1.17)	0.08	1.65 (1.48–1.82)	0.09	5.445	0.000
16	0.89 (0.79–1.00)	0.05	1.69 (1.54–1.83)	0.07	8.784	0.000
17	0.94 (0.81–1.07)	0.07	1.80 (1.62–1.99)	0.09	7.499	0.000
18	1.06 (0.84–1.28)	0.11	1.65 (1.44–1.87)	0.11	3.843	0.000
19	1.04 (0.74–1.34)	0.15	1.70 (1.42–1.98)	0.14	3.136	0.002

^‡^Estimated 95% confidence interval for the group mean

**Table 8:** Total outpatient costs, KVB data (estimates correspond to Fig. 2a)

Quarter	Control group		Observation group		Test of equal group means	
	Mean (95% CI)^‡^	S.E	Mean (95% CI)^‡^	S.E	*t* statistic	*p* value
0	221.44 (218.91–223.98)	1.29	248.50 (246.43–250.57)	1.06	16.215	0.000
1	147.91 (145.26–150.56)	1.35	144.55 (142.27–146.84)	1.17	− 1.879	0.060
2	135.77 (133.26–138.29)	1.29	129.46 (127.17–131.75)	1.17	− 3.632	0.000
3	134.95 (132.32–137.57)	1.34	129.43 (127.09–131.77)	1.19	− 3.077	0.002
4	135.99 (133.55–138.43)	1.25	129.65 (127.54–131.77)	1.08	− 3.843	0.000
5	131.57 (129.20–133.94)	1.21	128.19 (126.00–130.39)	1.12	− 2.048	0.041^†^
6	132.13 (129.65–134.61)	1.26	126.75 (124.61–128.89)	1.09	− 3.222	0.001
7	133.80 (131.31–136.28)	1.27	130.47 (128.19–132.74)	1.16	− 1.939	0.052
8	137.12 (134.58–139.66)	1.30	137.13 (134.85–139.41)	1.16	0.004	0.997
9	136.37 (133.61–139.13)	1.41	132.71 (130.43–135.00)	1.17	− 2.002	0.045^†^
10	136.12 (133.54–138.71)	1.32	133.12 (130.82–135.43)	1.18	-1.699	0.089
11	136.66 (134.07–139.26)	1.33	134.09 (131.72–136.46)	1.21	− 1.434	0.152
12	141.13 (138.46–143.80)	1.36	138.16 (135.75–140.57)	1.23	− 1.619	0.106
13	138.06 (135.43–140.69)	1.34	135.49 (133.17–137.82)	1.19	− 1.433	0.152
14	139.89 (137.22–142.55)	1.36	136.11 (133.78–138.45)	1.19	− 2.088	0.037^†^
15	141.83 (139.03–144.63)	1.43	138.34 (135.96–140.73)	1.22	− 1.860	0.063
16	143.46 (140.72–146.20)	1.40	140.71 (138.33–143.09)	1.21	− 1.487	0.137
17	142.22 (139.45–144.99)	1.41	140.76 (138.33–143.19)	1.24	− 0.778	0.437
18	140.36 (137.55–143.16)	1.43	136.67 (134.33–139.02)	1.20	− 1.973	0.048^†^
19	114.28 (111.67–116.88)	1.33	111.38 (109.17–113.59)	1.13	− 1.663	0.096

^‡^Estimated 95% confidence interval for the group mean

^†^Cost differential statistically significant at the 5% level, although 95% confidence intervals overlap

**Table 9:** Total outpatient costs, GWQ data (estimates correspond to Fig. 2b)

Quarter	Control group		Observation group		Test of equal group means	
	Mean (95% CI)^‡^	S.E	Mean (95% CI)^‡^	S.E	*t* statistic	*p* value
0	212.71 (206.66–218.75)	3.08	251.80 (246.17–257.43)	2.87	9.276	0.000
1	136.66 (130.23–143.09)	3.28	141.48 (136.53–146.43)	2.53	1.164	0.245
2	124.63 (117.97–131.28)	3.40	124.63 (118.10–131.16)	3.33	0.000	1.000
3	123.23 (117.29–129.18)	3.03	122.30 (117.15–127.44)	2.63	− 0.233	0.816
4	124.82 (118.62–131.02)	3.16	123.45 (118.29–128.60)	2.63	− 0.335	0.738
5	120.06 (114.34–125.78)	2.92	124.18 (118.49–129.87)	2.90	1.001	0.317
6	121.39 (115.44–127.34)	3.04	122.26 (117.08–127.44)	2.64	0.216	0.829
7	125.36 (119.57–131.15)	2.95	123.88 (118.83–128.93)	2.58	− 0.377	0.706
8	127.84 (121.99–133.68)	2.98	133.72 (128.63–138.81)	2.60	1.488	0.137
9	127.02 (121.12–132.92)	3.01	132.63 (126.85–138.40)	2.95	1.330	0.183
10	129.78 (123.58–135.97)	3.16	134.91 (129.48–140.34)	2.77	1.221	0.222
11	129.30 (123.59–135.01)	2.91	133.06 (127.47–138.65)	2.85	0.922	0.357
12	138.41 (131.98–144.83)	3.28	137.03 (131.26–142.80)	2.94	− 0.313	0.754
13	139.96 (132.75–147.17)	3.68	139.28 (133.36–145.21)	3.02	− 0.143	0.887
14	139.54 (132.35–146.72)	3.67	135.73 (129.60–141.86)	3.13	− 0.791	0.429
15	143.02 (136.04–149.99)	3.56	137.31 (131.86–142.77)	2.78	− 1.262	0.207
16	147.03 (139.01–155.06)	4.09	143.20 (136.89–149.50)	3.22	− 0.737	0.461
17	145.15 (136.92–153.38)	4.20	140.93 (134.44–147.41)	3.31	− 0.791	0.429
18	144.08 (134.96–153.19)	4.65	139.32 (132.42–146.21)	3.52	− 0.817	0.414
19	154.12 (139.14–169.07)	7.63	139.88 (131.49–148.27)	4.28	− 1.628	0.103

^‡^Estimated 95% confidence interval for the group mean

**Table 10:** Thyroid-specific inpatient costs, GWQ data (estimates correspond to Fig. 3a)

Quarter	Control group		Observation group		Test of equal group means	
	Mean (95% CI)^‡^	S.E	Mean (95% CI)^‡^	S.E	*t* statistic	*p* value
0	3.60 (1.11–6.10)	1.27	27.84 (20.22–35.46)	3.89	5.925	0.000
1	2.07 (0.08–4.05)	1.01	50.03 (39.64–60.43)	5.30	8.882	0.000
2	0.92 (-0.35–2.20)	0.65	19.64 (12.76–26.51)	3.51	5.245	0.000
3	0.73 (-0.26–1.73)	0.51	11.08 (6.53–15.63)	2.32	4.356	0.000
4	0.50 (-0.25–1.26)	0.39	7.59 (3.71–11.48)	1.98	3.514	0.000
5	3.02 (-0.69–6.74)	1.89	9.09 (4.13–14.05)	2.53	1.918	0.055
6	9.04 (1.17–16.90)	4.01	3.92 (1.21–6.63)	1.38	− 1.206	0.228
7	4.31 (0.15–8.47)	2.12	4.45 (1.48–7.42)	1.52	0.054	0.957
8	1.13 (-0.25–2.52)	0.71	3.38 (0.61–6.15)	1.41	1.421	0.155
9	4.46 (0.14–8.77)	2.20	9.81 (2.51–17.10)	3.72	1.237	0.216
10	3.37 (0.29–6.44)	1.57	5.30 (1.85–8.76)	1.76	0.820	0.412
11	19.83 (-4.58–44.24)	12.45	11.54 (3.93–19.15)	3.88	− 0.636	0.525
12	4.75 (0.02–9.48)	2.41	9.85 (2.64–17.06)	3.68	1.160	0.246
13	11.54 (0.47–22.61)	5.65	13.67 (-3.47–30.81)	8.75	0.204	0.838
14	8.29 (2.56–14.02)	2.92	3.74 (1.12–6.37)	1.34	− 1.413	0.158
15	4.88 (1.74–8.02)	1.60	6.15 (1.68–10.62)	2.28	0.456	0.648
16	14.00 (-3.47–31.47)	8.91	6.64 (2.28–10.99)	2.22	− 0.801	0.423
17	5.12 (0.44–9.80)	2.39	12.63 (4.39–20.88)	4.21	1.553	0.120
18	7.06 (1.80–12.32)	2.68	12.14 (3.62–20.66)	4.35	0.994	0.320
19	2.22 (-1.60–6.05)	1.95	3.79 (-1.39–8.97)	2.64	0.476	0.634

^‡^Estimated 95% confidence interval for the group mean

**Table 11:** Total inpatient costs, GWQ data (estimates correspond to Fig. 3b)

Quarter	Control group		Observation group		Test of equal group means	
	Mean (95% CI)^‡^	S.E	Mean (95% CI)^‡^	S.E	*t* statistic	*p* value
0	227.99 (187.91–268.07)	20.45	170.68 (145.81–195.55)	12.69	− 2.381	0.017^†^
1	224.06 (187.35–260.76)	18.73	257.80 (161.27–354.33)	49.25	0.640	0.522
2	184.46 (153.48–215.44)	15.81	166.38 (128.48–204.28)	19.34	− 0.724	0.469
3	177.41 (144.37–210.45)	16.86	166.65 (101.64–231.65)	33.17	− 0.289	0.772
4	171.29 (131.46–211.13)	20.32	144.17 (113.11–175.23)	15.85	− 1.052	0.293
5	141.99 (115.05–168.93)	13.75	146.29 (122.26–170.32)	12.26	0.233	0.816
6	187.85 (142.93–232.76)	22.92	130.96 (106.23–155.69)	12.62	− 2.174	0.030^†^
7	149.1 (122.76–175.44)	13.44	139.87 (112.64–167.10)	13.89	− 0.478	0.633
8	162.17 (132.65–191.68)	15.06	132.72 (108.95–156.49)	12.13	− 1.523	0.128
9	153.81 (127.93–179.69)	13.20	149.25 (124.73–173.77)	12.51	− 0.250	0.802
10	177.80 (147.16–208.44)	15.63	135.84 (112.82–158.86)	11.75	− 2.146	0.032^†^
11	209.11 (157.39–260.83)	26.39	152.72 (117.23–188.20)	18.11	− 1.762	0.078
12	172.08 (137.76–206.39)	17.51	137.06 (113.05–161.06)	12.25	− 1.639	0.101
13	179.33 (151.01–207.66)	14.45	204.77 (145.98–263.57)	30.00	0.764	0.445
14	208.15 (159.25–257.06)	24.95	179.04 (141.97–216.12)	18.91	− 0.930	0.353
15	223.80 (186.72–260.88)	18.92	144.13 (118.97–169.29)	12.84	− 3.485	0.000
16	241.21 (184.75–297.67)	28.81	153.74 (125.33–182.14)	14.49	− 2.712	0.007
17	243.78 (184.25–303.30)	30.37	153.52 (119.71–187.33)	17.25	− 2.584	0.010^†^
18	175.31 (134.95–215.68)	20.59	173.17 (130.33–216.02)	21.86	− 0.071	0.943
19	154.10 (112.00–196.19)	21.48	114.23 (84.65–143.81)	15.09	− 1.519	0.129

^‡^Estimated 95% confidence interval for the group mean

^†^Cost differential statistically significant at the 5% level, although 95% confidence intervals overlap

**Table 12:** Thyroid-specific pharmaceutical costs, GWQ data (estimates corresp. to Fig. 4a)

Quarter	Control group		Observation group		Test of equal group means	
	Mean (95% CI)^‡^	S.E	Mean (95% CI)^‡^	S.E	*t* statistic	*p* value
0	0.36 (0.29–0.43)	0.03	2.72 (2.55–2.89)	0.09	25.701	0.000
1	0.28 (0.23–0.34)	0.03	3.05 (2.87–3.24)	0.10	27.834	0.000
2	0.31 (0.25–0.37)	0.03	2.71 (2.54–2.87)	0.09	26.483	0.000
3	0.36 (0.30–0.42)	0.03	2.58 (2.42–2.74)	0.08	25.009	0.000
4	0.34 (0.28–0.40)	0.03	2.61 (2.44–2.77)	0.08	25.658	0.000
5	0.38 (0.31–0.44)	0.03	2.53 (2.37–2.68)	0.08	24.935	0.000
6	0.42 (0.35–0.48)	0.03	2.58 (2.42–2.74)	0.08	24.360	0.000
7	0.43 (0.37–0.50)	0.03	2.63 (2.47–2.78)	0.08	24.973	0.000
8	0.46 (0.39–0.53)	0.04	2.53 (2.38–2.69)	0.08	24.040	0.000
9	0.49 (0.42–0.56)	0.04	2.61 (2.46–2.77)	0.08	24.312	0.000
10	0.49 (0.42–0.57)	0.04	2.71 (2.55–2.87)	0.08	24.235	0.000
11	0.53 (0.46–0.61)	0.04	2.72 (2.56–2.88)	0.08	24.053	0.000
12	0.57 (0.49–0.65)	0.04	2.67 (2.52–2.83)	0.08	23.644	0.000
13	0.61 (0.53–0.69)	0.04	2.72 (2.56–2.87)	0.08	23.529	0.000
14	0.63 (0.55–0.71)	0.04	2.66 (2.51–2.81)	0.08	23.049	0.000
15	0.64 (0.56–0.72)	0.04	2.65 (2.49–2.80)	0.08	22.452	0.000
16	0.69 (0.61–0.78)	0.04	2.69 (2.54–2.84)	0.08	22.413	0.000
17	0.69 (0.59–0.78)	0.05	2.68 (2.51–2.85)	0.09	19.780	0.000
18	0.72 (0.60–0.83)	0.06	2.82 (2.60–3.03)	0.11	16.965	0.000
19	0.78 (0.61–0.95)	0.08	2.91 (2.62–3.19)	0.15	12.615	0.000

^‡^Estimated 95% confidence interval for the group mean

**Table 13:** Total pharmaceutical costs, GWQ data (estimates correspond to Fig. 4b)

Quarter	Control group		Observation group		Test of equal group means	
	Mean (95% CI)^‡^	S.E	Mean (95% CI)^‡^	S.E	*t* statistic	*p* value
0	82.92 (70.90–94.93)	6.13	62.76 (54.17–71.35)	4.38	− 2.675	0.007^†^
1	89.71 (73.58–105.84)	8.23	72.73 (58.90–86.57)	7.06	− 1.565	0.117
2	93.12 (71.99–114.25)	10.78	73.89 (59.70–88.09)	7.24	− 1.481	0.139
3	78.79 (67.84–89.73)	5.58	70.23 (58.31–82.16)	6.08	− 1.036	0.300
4	81.99 (70.11–93.88)	6.07	64.43 (54.11–74.74)	5.26	− 2.188	0.029^†^
5	82.93 (68.90–96.96)	7.16	67.54 (56.21–78.87)	5.78	− 1.672	0.094
6	80.24 (68.19–92.30)	6.15	69.91 (58.29–81.52)	5.93	− 1.210	0.226
7	77.79 (67.53–88.04)	5.23	65.40 (56.05–74.76)	4.77	− 1.749	0.080
8	85.84 (69.77–101.90)	8.20	76.23 (62.54–89.92)	6.98	− 0.892	0.372
9	85.06 (72.23–97.89)	6.55	80.24 (62.97–97.51)	8.81	− 0.439	0.661
10	81.54 (69.99–93.10)	5.90	74.37 (63.79–84.94)	5.40	− 0.898	0.369
11	100.40 (73.42–127.38)	13.77	90.53 (69.30–111.76)	10.83	− 0.564	0.573
12	96.65 (82.68–110.62)	7.13	87.03 (70.76–103.30)	8.30	− 0.880	0.379
13	105.48 (83.60–127.36)	11.16	83.07 (69.82–96.31)	6.76	− 1.717	0.086
14	101.52 (84.29–118.75)	8.79	95.50 (73.68–117.32)	11.13	− 0.424	0.671
15	106.88 (89.75–124.00)	8.74	86.46 (70.64–102.27)	8.07	− 1.717	0.086
16	125.40 (99.10–151.70)	13.42	79.67 (67.17–92.18)	6.38	− 3.078	0.002
17	127.27 (98.35–156.18)	14.75	83.59 (68.44–98.74)	7.73	− 2.623	0.009^†^
18	120.49 (87.53–153.45)	16.82	93.66 (72.61–114.71)	10.74	− 1.345	0.179
19	108.14 (79.06–137.21)	14.83	88.48 (65.47–111.49)	11.74	− 1.039	0.299

^‡^Estimated 95% confidence interval for the group mean

^†^Cost differential statistically significant at the 5% level, although 95% confidence intervals overlap

**Table 14:** Thyroid-specific costs, GWQ data (estimates correspond to Fig. 5a)

Quarter	Control group		Observation group		Test of equal group means	
	Mean (95% CI)^‡^	S.E	Mean (95% CI)^‡^	S.E	*t* statistic	*p* value
0	8.26 (5.76–10.76)	1.28	52.56 (44.79–60.34)	3.97	10.633	0.000
1	2.82 (0.83–4.82)	1.02	57.79 (47.28–68.31)	5.36	10.070	0.000
2	1.72 (0.44–3.00)	0.65	24.68 (17.75–31.62)	3.54	6.381	0.000
3	1.68 (0.68–2.68)	0.51	15.54 (10.92–20.15)	2.35	5.753	0.000
4	1.59 (0.78–2.40)	0.41	12.27 (8.32–16.22)	2.01	5.195	0.000
5	4.01 (0.29–7.73)	1.90	13.53 (8.52–18.53)	2.55	2.991	0.003
6	10.13 (2.25–18.00)	4.02	8.26 (5.49–11.03)	1.41	− 0.439	0.661
7	5.42 (1.25–9.60)	2.13	8.78 (5.77–11.79)	1.54	1.277	0.202
8	2.43 (1.02–3.83)	0.72	7.92 (5.11–10.73)	1.43	3.429	0.001
9	5.81 (1.49–10.13)	2.20	14.32 (7.00–21.63)	3.73	1.962	0.050
10	4.61 (1.52–7.69)	1.57	9.73 (6.26–13.21)	1.77	2.161	0.031^†^
11	21.19 (-3.22–45.60)	12.45	15.96 (8.30–23.56)	3.89	− 0.403	0.687^†^
12	6.25 (1.50–11.00)	2.42	14.09 (6.87–21.31)	3.68	1.778	0.075
13	13.07 (1.99–24.14)	5.65	18.16 (1.02–35.31)	8.75	0.489	0.625
14	9.85 (4.11–15.59)	2.93	7.91 (5.25–10.57)	1.36	− 0.602	0.547
15	6.54 (3.37–9.70)	1.61	10.45 (5.97–14.94)	2.29	1.398	0.162
16	15.59 (-1.89–33.06)	8.92	11.01 (6.65–15.38)	2.23	− 0.498	0.619
17	6.75 (2.03–11.46)	2.40	17.11 (8.85–25.38)	4.22	2.137	0.033
18	8.83 (3.52–14.15)	2.71	16.61 (8.05–25.17)	4.37	1.513	0.130^†^
19	4.05 (0.19–7.90)	1.97	8.39 (3.18–13.60)	2.66	1.314	0.189

^‡^Estimated 95% confidence interval for the group mean

^†^Cost differential statistically significant at the 5% level, although 95% confidence intervals overlap

**Table 15:** Total costs, GWQ data (estimates correspond to Fig. 5b)

Quarter	Control group		Observation group		Test of equal group means	
	Mean (95% CI)^‡^	S.E	Mean (95% CI)^‡^	S.E	*t *statistic	*p* value
0	523.61 (479.29–567.94)	22.61	485.24 (457.31–513.17)	14.25	− 1.436	0.151
1	450.43 (407.75–493.11)	21.78	472.02 (372.05–571.99)	51.01	0.389	0.697
2	402.21 (362.66–441.76)	20.18	364.90 (322.55–407.26)	21.61	− 1.262	0.207
3	379.43 (342.41–416.44)	18.89	359.18 (292.47–425.88)	34.03	− 0.520	0.603
4	378.11 (335.33–420.90)	21.83	332.04 (297.71–366.37)	17.52	− 1.646	0.100
5	344.98 (312.65–377.32)	16.50	338.01 (309.49–366.53)	14.55	− 0.317	0.751
6	389.48 (341.55–437.41)	24.46	323.13 (293.75–352.51)	14.99	− 2.313	0.021^†^
7	352.25 (322.21–382.29)	15.33	329.16 (298.98–359.33)	15.40	− 1.063	0.288
8	375.84 (340.52–411.16)	18.02	342.67 (313.40–371.94)	14.94	− 1.417	0.156
9	365.9 (334.61–397.18)	15.96	362.12 (329.60–394.64)	16.59	− 0.164	0.870
10	389.12 (354.07–424.17)	17.89	345.11 (317.82–372.41)	13.92	− 1.941	0.052
11	438.82 (378.87–498.77)	30.59	376.31 (333.16–419.46)	22.02	− 1.659	0.097
12	407.14 (367.73–446.54)	20.10	361.12 (329.63–392.60)	16.06	− 1.788	0.074
13	424.77 (385.62–463.93)	19.98	427.12 (365.66–488.58)	31.36	0.063	0.950
14	449.21 (395.65–502.76)	27.32	410.27 (364.87–455.67)	23.16	− 1.087	0.277
15	473.69 (430.29–517.10)	22.14	367.90 (335.76–400.03)	16.40	− 3.840	0.000
16	513.64 (448.98–578.31)	32.99	376.61 (343.44–409.77)	16.92	− 3.696	0.000
17	516.20 (445.19–587.20)	36.23	378.03 (338.54–417.52)	20.15	− 3.333	0.001
18	439.89 (384.45–495.32)	28.28	406.15 (356.38–455.91)	25.39	− 0.888	0.375
19	416.36 (353.67–479.05)	31.98	342.59 (300.65–384.53)	21.40	− 1.917	0.055

^‡^Estimated 95% confidence interval for the group mean

^†^Cost differential statistically significant at the 5% level, although 95% confidence intervals overlap

**Table 16:** Thyroid-specific outpatient costs, KVB alternative matching (estimates correspond to Fig. 6b)

Quarter	Control group		Observation group		Test of equal group means	
	Mean (95% CI)^‡^	S.E	Mean (95% CI)^‡^	S.E	*t* statistic	*p* value
0	4.95 (4.86–5.04)	0.05	18.20 (18.00–18.39)	0.10	121.193	0.000
1	1.06 (0.99–1.14)	0.04	1.95 (1.84–2.05)	0.05	13.571	0.000
2	0.75 (0.69–0.80)	0.03	1.34 (1.27–1.42)	0.04	12.800	0.000
3	0.69 (0.64–0.74)	0.02	1.18 (1.12–1.24)	0.03	12.145	0.000
4	0.76 (0.72–0.81)	0.02	1.28 (1.21–1.34)	0.03	12.586	0.000
5	0.75 (0.70–0.80)	0.03	1.19 (1.13–1.25)	0.03	10.602	0.000
6	0.79 (0.73–0.84)	0.03	1.14 (1.08–1.20)	0.03	8.881	0.000
7	0.77 (0.72–0.82)	0.03	1.25 (1.19–1.31)	0.03	11.945	0.000
8	0.88 (0.83–0.93)	0.03	1.66 (1.59–1.73)	0.04	17.590	0.000
9	0.73 (0.69–0.78)	0.02	1.46 (1.39–1.53)	0.04	17.463	0.000
10	0.80 (0.75–0.85)	0.03	1.25 (1.19–1.31)	0.03	11.289	0.000
11	0.80 (0.75–0.85)	0.03	1.24 (1.17–1.30)	0.03	10.182	0.000
12	0.85 (0.80–0.90)	0.03	1.32 (1.25–1.38)	0.03	11.285	0.000
13	0.83 (0.77–0.88)	0.03	1.26 (1.20–1.32)	0.03	10.329	0.000
14	0.82 (0.77–0.87)	0.03	1.28 (1.22–1.35)	0.03	11.137	0.000
15	0.90 (0.84–0.95)	0.03	1.27 (1.20–1.33)	0.03	8.650	0.000
16	0.91 (0.86–0.97)	0.03	1.48 (1.41–1.55)	0.03	12.632	0.000
17	0.92 (0.87–0.98)	0.03	1.39 (1.33–1.46)	0.03	10.808	0.000
18	0.87 (0.81–0.92)	0.03	1.30 (1.24–1.37)	0.03	10.146	0.000
19	0.87 (0.81–0.92)	0.03	1.29 (1.23–1.35)	0.03	9.968	0.000

^‡^Estimated 95% confidence interval for the group mean

**Table 17:** Total outpatient costs, KVB alternative matching (estimates corresp. to Fig. 7b)

Quarter	Control group		Observation group		Test of equal group means	
	Mean (95% CI)^‡^	S.E	Mean (95% CI)^‡^	S.E	*t* statistic	*p* value
0	226.78 (223.24–230.32)	1.80	242.81 (240.04–245.58)	1.41	6.995	0.000
1	148.24 (144.60–151.88)	1.86	136.50 (133.48–139.52)	1.54	− 4.867	0.000
2	135.01 (131.63–138.39)	1.72	123.72 (120.62–126.82)	1.58	− 4.825	0.000
3	132.05 (128.64–135.45)	1.74	125.83 (122.62–129.03)	1.63	− 2.607	0.009^†^
4	134.42 (131.16–137.68)	1.66	127.53 (124.62–130.44)	1.49	− 3.091	0.002
5	130.70 (127.57–133.83)	1.60	127.14 (124.14–130.14)	1.53	− 1.609	0.108
6	130.63 (127.27–134.00)	1.72	125.95 (122.95–128.94)	1.53	− 2.041	0.041^†^
7	130.89 (127.66–134.11)	1.65	129.71 (126.63–132.79)	1.57	− 0.516	0.606
8	135.54 (132.13–138.94)	1.74	138.99 (135.74–142.24)	1.66	1.439	0.150
9	131.46 (128.15–134.78)	1.69	132.49 (129.34–135.64)	1.61	0.439	0.661
10	133.04 (129.78–136.31)	1.66	132.39 (129.06–135.72)	1.70	− 0.276	0.783
11	134.76 (131.31–138.22)	1.76	133.11 (129.79–136.44)	1.70	− 0.675	0.500
12	139.41 (135.98–142.84)	1.75	139.51 (136.14–142.88)	1.72	0.040	0.968
13	134.59 (131.26–137.91)	1.70	136.27 (133.09–139.45)	1.62	0.717	0.473
14	137.87 (134.37–141.37)	1.79	138.15 (134.72–141.59)	1.75	0.112	0.911
15	136.44 (133.15–139.74)	1.68	139.09 (135.75–142.43)	1.70	1.106	0.269
16	139.86 (136.49–143.23)	1.72	142.94 (139.43–146.44)	1.79	1.240	0.215
17	140.32 (136.77–143.88)	1.81	143.65 (140.07–147.23)	1.83	1.292	0.196
18	136.37 (132.92–139.83)	1.76	137.03 (133.64–140.42)	1.73	0.267	0.790
19	111.41 (107.94–114.88)	1.77	111.44 (108.27–114.61)	1.62	0.013	0.990

^‡^Estimated 95% confidence interval for the group mean

^†^Cost differential statistically significant at the 5% level, although 95% confidence intervals overlap

**Table 18:** Alternative matching: pre-treatment means of observation and control group and standardized bias (KVB data only)

	Unmatched		Matched		Standardized bias %	
	Observation	Control	Observation	Control	Unmatched	Matched
Age	48	49.1	48	48.1	6.35	0.61
Female	0.60	0.54	0.60	0.60	12.16	0.00
No indication	0.60	0.60	0.60	0.60	1.62	0.00
# InBA = 0	0.10	0.08	0.10	0.10	7.91	0.65
# InBA = 1	0.06	0.05	0.06	0.06	4.39	0.85
# InBA = 2	0.07	0.07	0.07	0.07	2.38	0.39
# InBA ≥ 3	0.77	0.80	0.77	0.77	9.48	0.24
N	36,120	629,006	36,120	36,120		

## Footnotes

1 It is important to note that—using this definition—for a given patient, one cannot determine whether he or she is subject to overdiagnosis since one does not know the counterfactual medical outcome on the individual level. Providing ‘too many’ diagnostic services might be accompanied by another problem related to but still distinct from overdiagnosis, namely an increased number of false positive test results that may also harm patients.

2 Nowadays “company-based health insurance” is little more than a label; since 1996 any insurant is allowed to contract with company-based sickness fund, irrespective of whether or not any relation to the respective company exists.

3 This would not be possible for data protection reasons, even if the overlap of the data would be bigger.

4 See Table 2 in the appendix for an overview of exclusion criteria, which include thyroid-specific outpatient services and diagnosis in the GWQ data, as well as the KVB data. For the GWQ data, we additionally excluded patients who received thyroid-specific inpatient services.

5 See Table 3 in the appendix for an overview of implausible diagnoses which led to an exclusion from the sample.

6 It may happen that the sonography did not take place until January 2013, when the TSH test was taken in December 2012. If someone has received several TSH tests and a sonography was performed only after the second TSH test or later he/she is not included in the sample.

7 See Tables 4 and 5 in the appendix for the outputs of the logistic regressions.

8 Thyroid-specific diagnosis/relevant complaints include: fatigue (R53, T73.3, G93.3, F48.0,F43), sleep disorder (G47.0), globe/swallowing disorder (R13), hypertension (I10), weight loss (R63, R64) and sweating (R61.1), each including all n-digit ICD codes.

9 The standardized bias in percent $100\bullet {\left({0.5Var\left({x}_{o}\right)}+{0.5Var\left({x}_{c}\right)}\right)}^{-0.5}\bullet \left({\overline{x} }_{o}-{\overline{x} }_{c}\right)$ represents the mean difference of the observation ($o$) and control ($c$) group for each covariate ($x$) as a percentage of the square root of the average of the sample variance.

10 Because of the large sample sizes, for statistical inference one can rely on asymptotic normality of the group means. This likewise applies to relying on *t* statistics for formally testing equality of group means.

11 The shares of thyroid-specific costs in total costs are visualized in Fig. 8 in the Appendix.

12 Which according to EBM code 33012 amounts to € 8.46.

13 We consider a case and the associated costs thyroid-specific, if thyroid-specific OPS codes and/or a thyroid-related discharge diagnosis was coded for that case.

14 With regard to pharmaceutical costs thyroid-specific refers to the ATC Codes H03A, H03B, and H03C.

## Code availability

The SQL and Stata^®^ code used for analyzing the data can be made available.
